# Lack of immunogenicity for an influenza‐derived peptide across the HLA‐B44 supertype molecules

**DOI:** 10.1002/cti2.70051

**Published:** 2025-09-19

**Authors:** Samuel Liwei Leong, Janesha C Maddumage, Stephanie Gras, Emma J Grant

**Affiliations:** ^1^ Infection and Immunity Program, La Trobe Institute for Molecular Science (LIMS) La Trobe University Bundoora VIC Australia; ^2^ Department of Biochemistry and Chemistry, School of Agriculture, Biomedicine and Environment (SABE) La Trobe University Bundoora VIC Australia; ^3^ Department of Biochemistry and Molecular Biology Monash University Clayton VIC Australia

**Keywords:** CD8^+^ T cells, HLA‐B44 supertype, HLA‐I supertype, immunogenicity, influenza, structural prediction

## Abstract

**Objectives:**

CD8^+^ T cells are protective against influenza and there is an interest in designing a future CD8^+^ T‐cell‐mediated vaccine. However, a significant challenge is the extensive polymorphism of Human Leukocyte Antigen class I (HLA‐I) molecules, the targets of CD8^+^ T cells. Despite this, HLA supertypes have been defined as a subset of HLA‐I molecules sharing similar peptide motif preferences that may present overlapping peptide repertoires. Therefore, selecting immunogenic peptides presented by a range of HLA‐I molecules for inclusion in a vaccine may partially overcome the challenge presented by HLA‐I polymorphism.

**Methods:**

In this study, we investigated the presentation and immunogenicity of a known HLA‐B*44:03‐restricted influenza‐derived peptide NS1_195‐203_ across the HLA‐B44 supertype. Using TFold and AlphaFold2, we predicted the structures of the NS1_195‐203_ bound by the HLA‐B44 supertype molecules, including HLA‐B*44:02, HLA‐B*44:03, HLA‐B*40:01, HLA‐B*40:01 and HLA‐B*45:01. Peripheral blood mononuclear cells (PBMCs) isolated from donors expressing one of these HLA‐B44 supertype molecules were used to generate CD8^+^ T‐cell lines against the NS1_195‐203_ peptide and assess its immunogenicity via intracellular cytokine staining assay.

**Results:**

The structures predicted with TFold and AlphaFold2 of the NS1_195‐203_ peptide in complex with the HLA‐B44 allomorphs were overall similar, with some notable differences at the peptide P9‐Trp. A polyfunctional NS1_195‐203_‐specific CD8^+^ T‐cell response was observed in HLA‐B*44:03^+^ and HLA‐B*44:02^+^ samples; however, minimal responses were observed in the three other HLA‐B44^+^ supertype molecules.

**Conclusion:**

Although HLA molecules from the same supertype may be able to present the same peptide, this will not always result in CD8^+^ T‐cell responses. As such, HLA‐I supertypes, defined based on peptide binding motif and presentation, do not include information on immunogenicity and are not currently able to be used on their own to select epitopes as vaccine candidates. However, new knowledge on HLA supertypes may help curate sets of peptides that are potential vaccine targets and applicable to a range of HLA allomorphs.

## Graphical abstract

Focussing on HLA‐I molecules grouped into HLA‐I supertypes may help relieve the bottleneck in selecting peptides presented by a range of HLA‐I molecules for CD8+ T cell mediated vaccine design. We investigated this by focussing on a known immunogenic HLA‐B*44:03‐restricted influenza‐derived peptide, namely NS1195‐203. Using TFold and AlphaFold, we predicted the structure of several HLA‐B44 supertype molecules presenting this peptide. We also found that this peptide was immunogenic in cells derived from individuals with HLA‐B*44:03, HLA‐B*44:02 and to a lesser extent HLA‐B*40:01.

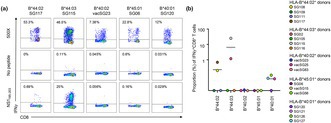

## Introduction

Influenza virus continues to cause significant morbidity and mortality annually, with more than 650 000 individuals worldwide succumbing to seasonal infections despite vaccines being widely available.^1^ Many licensed influenza vaccines mainly contain the surface glycoprotein haemagglutinin (HA) that is highly susceptible to antigenic drift mandating that vaccines are updated and administered annually.^2^, ^3^ As such, the need for novel vaccines is required.^1^, ^2^ CD8^+^ T cells are well known for their ability to provide protection against several pathogens, including the influenza virus, and there is a great interest in developing a CD8^+^ T cell‐mediated vaccine against influenza.^2^, ^4^, ^5^, ^6^ CD8^+^ T cells typically target peptides derived from internal proteins such as matrix, polymerase basic and non‐structural proteins, which tend to be more conserved than the surface HA glycoprotein, making them an attractive target.^2^, ^7^, ^8^ CD8^+^ T cells recognise Human Leukocyte Antigen class I (HLA‐I) molecules that present short protein‐derived peptides.^9^ HLA‐I molecules are genetically encoded and highly polymorphic, with over 28 000 *HLA‐I* alleles described to date.^10^ Moreover, each *HLA‐I* allele is expressed across the global population disproportionally.^11^ For example, it is estimated that 5.4% of individuals express the HLA‐B*35:01 molecule whereas only 3.4% of the global population express HLA‐B*15:01.^11^ HLA‐I molecules also have unique peptide binding pockets that dictate their preferences to different residues.^9^ This is based on the amino acid sequences of the bound peptides typically at position 2 (P2) and the last position (PΩ), which anchor the peptide into the HLA‐I binding cleft.^9^ Traditionally, research on CD8^+^ T cell‐mediated vaccines has concentrated on several peptides presented by a single HLA‐I molecule. For instance, the HLA‐A*02:01‐focussed Flu‐V vaccine which recently concluded phase IIb clinical trials only provides protection to a limited percentage of the population (15.2%).^11^, ^12^, ^13^

One way to cover large proportions of the global population with a vaccination that contains a limited set of peptides or proteins containing epitopes that cover a range of HLA‐I molecules is to consider HLA supertypes. HLA‐I molecules have been classified into HLA supertypes based on having similar peptide binding motifs or other structural characteristics important for peptide presentation.^14^, ^15^, ^16^, ^17^ Importantly, the classification mostly considers whether HLA‐I molecules can, or are likely to, present a peptide but does not consider its ability to stimulate a CD8^+^ T‐cell response. There have been several rearrangements of HLA‐I into supertypes over the decades including classifications based on peptide motifs,^16^ conservation of key residues interacting with the peptides^15^ and more recently, structures of peptide‐HLA complexes.^17^ The 2014 classification by Harjanto *et al*,^15^ which considers HLA classification based on important peptide residues, was used as the basis of this study. There are 11 HLA‐I supertypes, including five HLA‐A and six HLA‐B supertypes.^15^ One of the HLA‐B supertypes is the HLA‐B44 supertype that includes HLA‐B*40:01, HLA‐B*40:02, HLA‐B*44:02, HLA‐B*44:03 and HLA‐B*45:01 allomorphs.^15^ The total cumulative distribution of these allomorphs equates to ~16.9% or 1/6th of the global population, or approximately 1.3 billion individuals.^11^ Thus, focusing only on a few highly frequent allomorphs representative of HLA supertypes will enable global coverage. Interestingly, three of these five HLA‐I allomorphs are also within the top 10 *HLA‐B* alleles expressed worldwide by frequency.^2^ Moreover, together these five HLA‐I molecules are well represented in many continents, including Europe, Africa, North America, South America and Oceania.^11^

In this study, we investigated CD8^+^ T‐cell immunogenicity towards a peptide presented by HLA‐B44 supertype molecules. We selected the known immunogenic HLA‐B*44:03‐restricted SETLQRFAW peptide derived from the influenza non‐structural 1 protein (hereafter referred to as NS1_195–203_). A previous study showed that the NS1_195–203_ stimulated > 70% of CD8^+^ T cells to produce IFNγ in CD8^+^ T‐cell lines.^18^ Moreover, NS1_195‐203_ is conserved in 100% of A/H1N1 and 99% of H3N2 variants circulating in Australia between 1971 and 2021,^18^ making it an attractive vaccine candidate if it induced CD8^+^ T‐cell responses in individuals with other HLA‐B44 allomorphs. Using NetMHCpan4.1, we first predicted the binding affinity of NS1_195‐203_ for each HLA‐I molecule. HLA‐B*44:02 and HLA‐B*44:03 were predicted to be strong binders of the NS1_195‐203_ peptide, while HLA‐B*40:01, HLA‐B*40:02 and HLA‐B*45:01 were predicted to be weak binders.^19^ We then used *in silico* modelling with TFold^20^ and AlphaFold2^21^ to predict each HLA‐B44‐NS1_195‐203_ structure. In all models, the peptide was binding canonically, with P2 and P9 acting as anchor residues. The predictions generated by both TFold^20^ and AlphaFold2^21^ gave similar structures at the exception of HLA‐B*44:03. Finally, we assessed the immunogenicity of the NS1_195‐203_ peptide in CD8^+^ T cells derived from samples expressing one of the HLA‐B44 supertype molecules using an intracellular cytokine staining (ICS) assay. The NS1_195‐203_ peptide was immunogenic in CD8^+^ T cells derived from donors expressing HLA‐B*44:03 or HLA‐B*44:02, with a higher IFNγ production in HLA‐B*44:03^+^ samples. Finally, limited CD8^+^ T‐cell responses were identified in samples expressing HLA‐B*40:01, but not in HLA‐B*40:02^+^ or HLA‐B*45:01^+^ samples tested.

In summary, this evidence suggests HLA molecules belonging to the same supertype may bind to the same peptide; however, HLA polymorphism can influence the way the peptide is presented, in turn affecting the T‐cell responses.

## Results

### NS1_195_ _‐203_ is predicted to be a strong binder for HLA‐B*44:02 and HLA‐B*44:03 molecules

**Figure 1:**
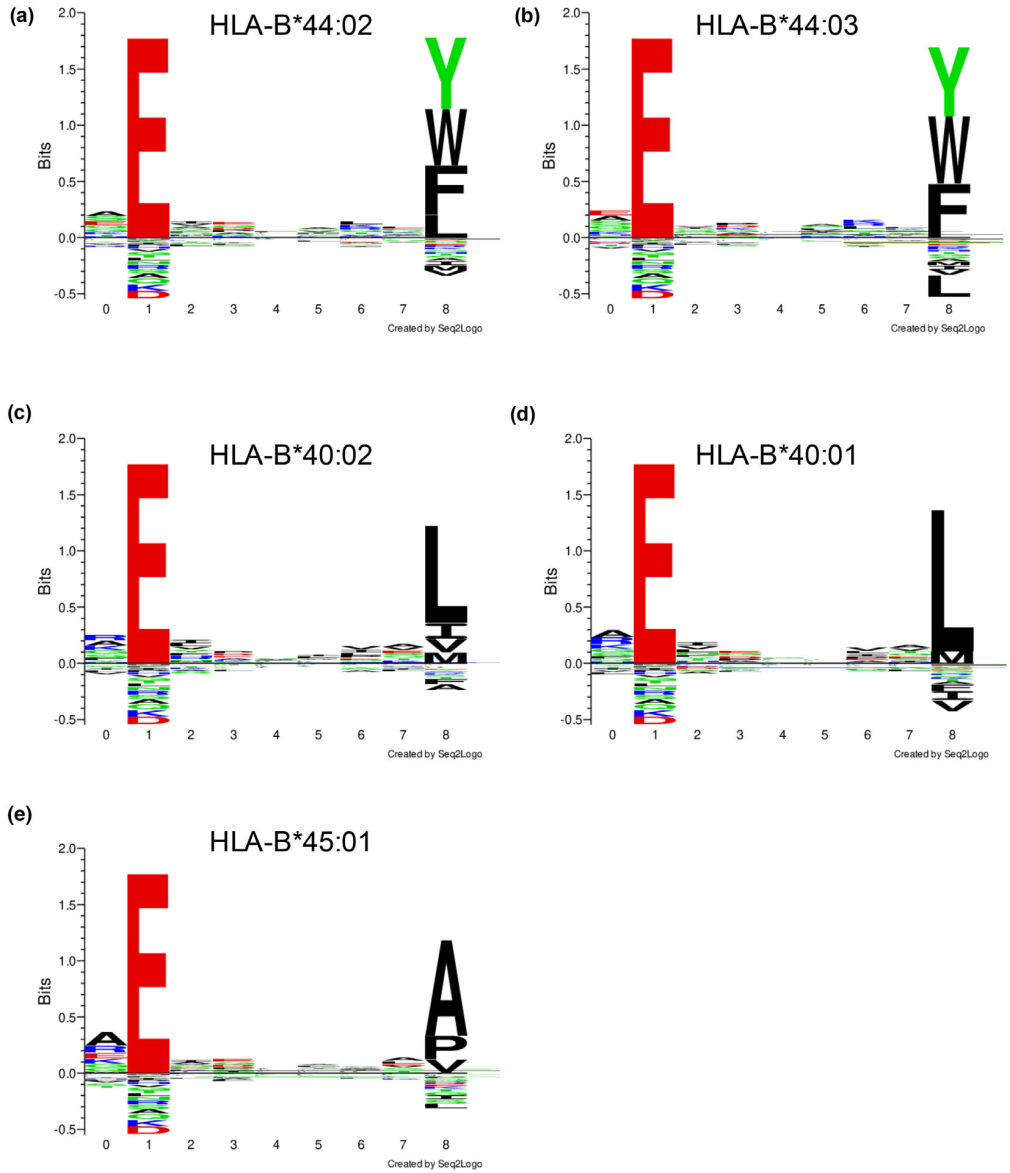
Predicted binding affinity of NS1_195–203_ to each HLA‐B44 supertype molecule and their peptide motif preferences. Peptide binding motif preferences for **(a)** HLA‐B*44:02, **(b)** HLA‐B*44:03, **(c)** HLA‐B*40:02, **(d)** HLA‐B*45:01 and **(e)** HLA‐B*40:01 obtained from NetMHCpan4.1.^19^

To investigate the potential presentation of the known HLA‐B*44:03‐restricted influenza‐derived peptide NS1_195‐203_ across the HLA‐B44 supertype, we first assessed their preferred peptide binding motifs (Figure 1). A sequence alignment of HLA‐B44 allomorphs shows that their B pocket is well conserved with only one polymorphic residue (Supplementary figure 1), while the F pocket had five residues that differ among the allomorphs (Supplementary figure 1). All five HLA‐B44 supertype allomorphs shared a preference for a Glutamic acid residue at position 2 (Figure 1), which is present in the NS1_195‐203_ peptide. HLA‐B*44:02 and HLA‐B*44:03 both have preferences for amino acids with large aromatic side chains (Y, W and F) at PΩ (Figure 1a and b), and the NS1_195‐203_ peptide harbours a PΩ‐Trp. NetMHCpan4.1 showed that these two allomorphs are predicted to have high affinity (< 15 nm) for the NS1_195‐203_ peptide (S**E**TLQRFA**W**), which fits within the preferred motif of these HLA‐I molecules (Table 1). Despite belonging to the same HLA‐I supertype as HLA‐B*44:03, the preference at PΩ for HLA‐B*40:01 and HLA‐B*40:02 is for a smaller hydrophobic residue (PΩ‐Leu), and an even smaller one for HLA‐B*45:01 with PΩ‐Ala (Figure 1c–e). These three allomorphs are predicted to weakly bind the NS1_195‐203_ peptide (Table 1), likely because of the presence of a large PΩ‐Trp that does not fit their preferred peptide binding motif.

**Table 1:** Predicted affinity and *in silico* score of the NS1_195‐203_ peptide for the B44 supertype molecules

HLA molecule	Predicted affinity (nm)	TFold error score	AlphaFold2 pLDDT
B*44:02	10.76	5.84	92.9
B*44:03	12.42	5.96	92.9
B*40:01	3069.3	7.65	91.1
B*40:02	921	9.49	93.2
B*45:01	2960.3	12.21	92.3

Predicted affinity of the NS1_195‐203_ peptide with each of the HLA‐B44 supertype molecules was determined using NetMHCpan‐4.1. The threshold for a strong peptide binder was < 0.5, a weak peptide binder was 0.5–2 and > 2 as a non‐binder.^19^ The TFold error score^20^ and AlphaFold2^21^ global pLDDT values were calculated to evaluate the structural confidence of the predicted models. The TFold score is based solely on the HLA cleft model prediction, while the AlphaFold2 pLDDT score applies to the entire model. Each AlphaFold score is reported as a cumulative value for both the HLA heavy chain and the peptide, excluding the β2m. For AlphaFold2 prediction, a high confidence score is a pLDDT > 90 and for TFold > 6.8.

### AlphaFold2 and TFold structural prediction of NS1_195_ _‐203_ presented by HLA‐B44 supertype

**Figure 2:**
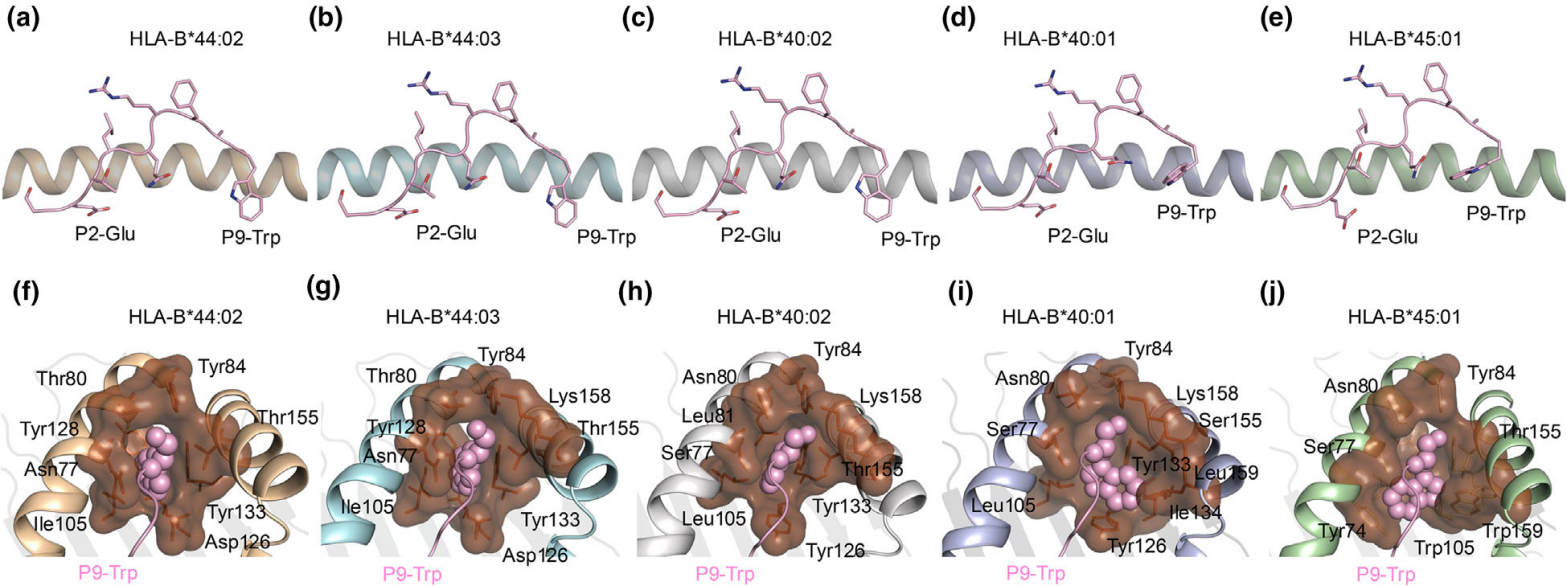
TFold predicted models of the NP_195‐203_ peptide presented by the HLA‐B44 supertype molecules. TFold predicted structure of the NS1_195–203_ peptide (pink cartoon and sticks) presented by **(a)** HLA‐B*44:02 (orange), **(b)** HLA‐B*44:03 (cyan), **(c)** HLA‐B*40:02 (grey), **(d)** HLA‐B*40:01 molecule (light blue) and **(e)** HLA‐B*45:01 (green). **(f–j)** Surface representation of the F pocket (brown) shown for each HLA allomorph. The primary anchor residue P9–Trp of NS1_195–203_ peptide is represented as pink spheres nested into the F pocket surrounded by HLA α1‐helix and α2‐helix.

To understand how the HLA‐B44 supertype allomorphs would present the NS1_195‐203_ peptide, we used TFold^20^ and AlphaFold2^21^ to predict the peptide‐HLA structures.^20^, ^21^ TFold is based on AlphaFold2 but specialised for HLA structures,^20^ and we have observed better prediction compared to crystal structures than with AlphaFold2.^22^ The five predicted structures shared the same HLA fold, showing how potentially the NS1_195‐203_ peptide binds to the allomorphs (Figure 2). The predicted pHLA structures were similar between TFold and AlphaFold2, with a root mean square deviation (r.m.s.d.) of 0.25–0.45 Å for the antigen binding cleft (Cα atoms of HLA residues 1–180) and 0.44–0.65 Å for the peptide (Cα atoms). The exception was the HLA‐B*44:03‐NS1_195‐203_, for which there was a clear difference in the peptide conformation with a r.m.s.d. of 1.64 Å (Supplementary figure 2), despite a similar HLA cleft model (r.m.s.d. = 0.25 Å). The TFold analysis assigned a score to each prediction (Table 1), and as indicated by Mikhaylov *et al*, TFold assigns a confidence score, with values below 6.8 indicating high predicted accuracy, though this may have some exceptions. The scores were lowest for HLA‐B*44:02 and HLA‐B*44:03 models (5.84 and 5.96, respectively) suggesting that the confidence in the predicted structures is higher for those allomorphs (Table 1).

**Figure 3:**
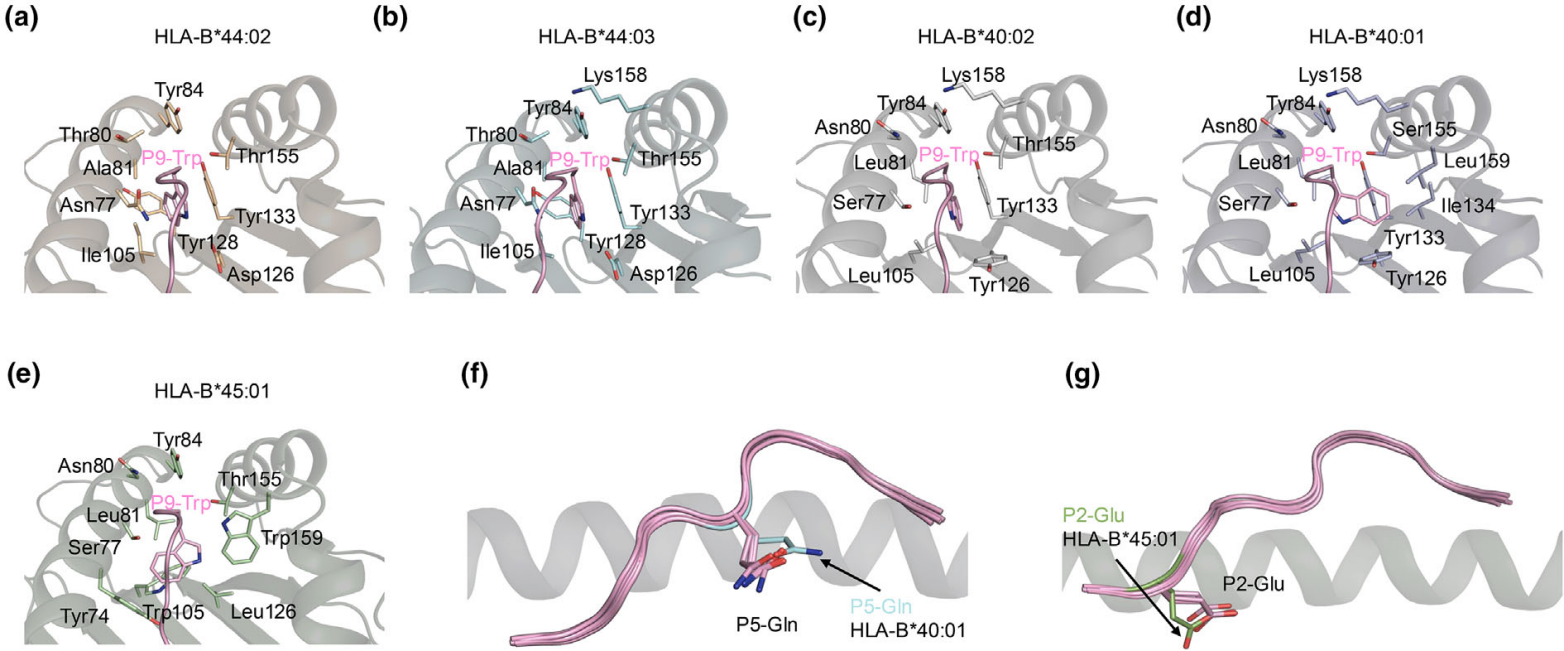
TFold predicted models of HLA‐B44 supertype molecules presenting the NS1_195–203_ peptide. TFold predicted structure of NS1_195–203_ peptide (pink stick) presented by **(a)** HLA‐B*44:02 (orange cartoon), **(b)** HLA‐B*44:03 (cyan cartoon), **(c)** HLA‐B*40:02 (grey cartoon), **(d)** HLA‐B*40:01 (light blue cartoon) and **(e)** HLA‐B*45:01 (green cartoon). **(f)** In HLA‐B*40:01 *the P5‐Gln side chain of the* NS1_195–203_ peptide *(cyan stick) adopts a different conformation compared to the P5‐Gln conformation in other supertypes (P5‐Gln of other supertypes are shown as pink sticks)*. **(g)** P2‐Glu of HLA‐B*45:01 (green stick) adopts a different conformation compared to other predicted supertypes (P2‐Glu *of other supertypes are shown as* pink sticks).

The models of the NS1_195‐203_ peptide backbone adopted a canonical conformation within the cleft of the HLA allomorphs,^9^ with anchor residues P2‐Glu and P9‐Trp binding to the B and F pockets, respectively (Figure 2). However, the P9‐Trp side chain conformation was different in each allomorph (Figure 2 and Supplementary figure 3). In the predicted structures of HLA‐B*44:02, HLA‐B*44:03 and HLA‐B*40:02, the P9‐Trp side chain was pointing down to the cleft, acting as a canonical anchor residue (Figure 3a–c). The P9‐Trp side chain adopted the same conformation in both HLA‐B*44:02 and HLA‐B*44:03 (Figure 3a and b) and slightly changed position in HLA‐B*40:02 (Figure 3c) to avoid steric clashes with the larger Leu81 instead of Ala81 in the two other allomorphs. In contrast, in HLA‐B*40:01 and HLA‐B*45:01, the P9‐Trp side chain turned 90° in the F pocket (Figure 3d and e, respectively), not following a canonical anchor residue conformation in those allomorphs. In HLA‐B*40:01, the P9‐Trp side chain is facing towards the HLA α2‐helix to avoid clashes with Leu81 and Tyr126 (Figure 3d). In HLA‐B*45:01, the P9‐Trp side chain is pointing towards the HLA α1‐helix to avoid clashes with Leu126, Trp159 and Trp105 that is directly underneath the P9‐Trp (Figure 3e). The predicted structures analysis reveals some differences in the rotamers conformation of P5‐Gln of HLA‐B*40:01 and the P2‐Glu of HLA‐B*45:01 compared to the other HLA allomorph models (Figure 3f and g).

### Not all HLA‐B44 allomorphs presenting the NS1_195_ _‐203_ peptide induce CD8 ^+^ T‐cell responses

**Figure 4:**
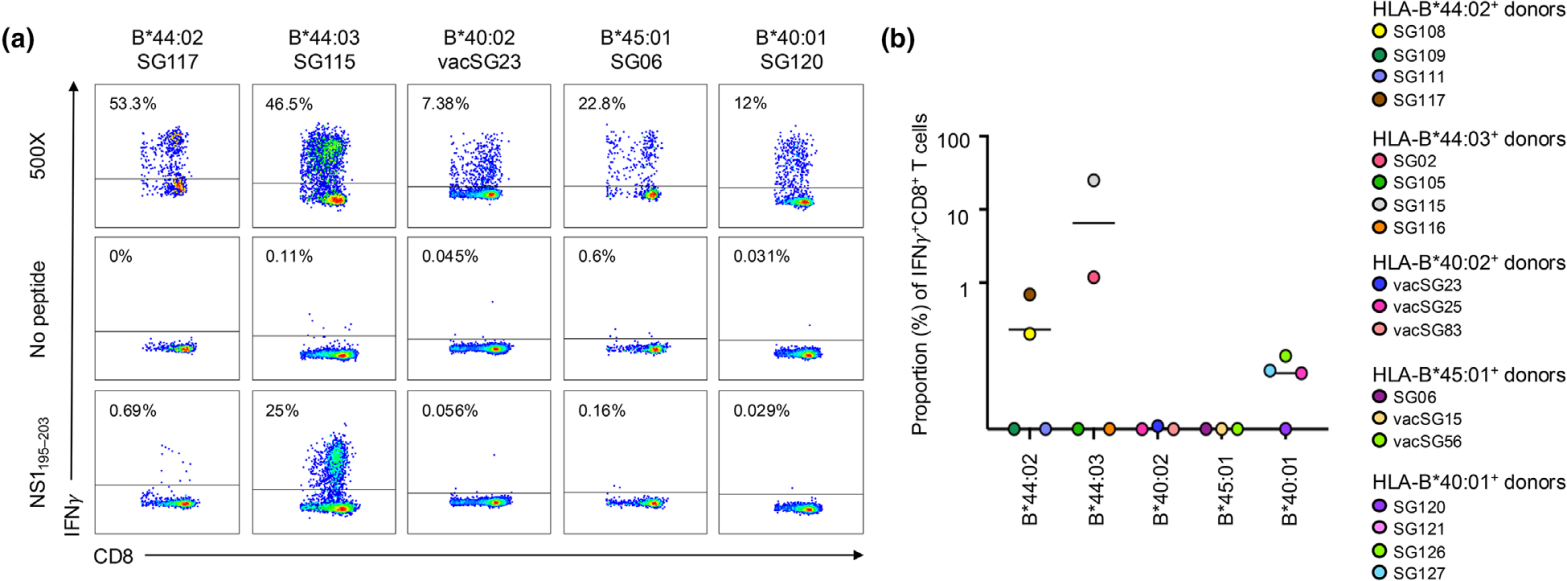
Intracellular cytokine staining of NS1_195–203_‐specific T‐cell lines determining immunogenicity for each HLA‐B44 supertype. PBMCs from HLA‐B*40:01^+^ (*n* = 4), HLA‐B*40:02^+^ (*n* = 3), HLA‐B*44:02^+^ (*n* = 4), HLA‐B*44:03^+^ (*n* = 4) and HLA‐B*45:01^+^ (*n* = 3) samples were stimulated with 10 μm of the NS1_195–203_ peptide for 10 days. On Day 10, an intracellular cytokine staining (ICS) assay was completed by restimulating with either the NS1_195–203_ peptide, no peptide (negative control) or 500× (positive control) to assess CD8^+^ T‐cell responses. **(a)** Representative FACS plots of IFNγ production by CD8^+^ T cells from donors with expressing B44 supertype molecules. **(b)** A scatterplot displaying the percentage of CD8^+^ T cells from donors expressing each of the HLA‐B44 allomorphs producing IFNγ (subtracted from no peptide control). The horizontal bar denotes the average (mean) and each coloured dot represents an individual donor.

As all five allomorphs might be able to present the NS1_195‐203_ peptide, we next wonder if they could induce a CD8^+^ T‐cell response. PBMCs isolated from HLA‐B*40:01^+^ (*n* = 4), HLA‐B*40:02^+^ (*n* = 3), HLA‐B*44:02^+^ (*n* = 4), HLA‐B*44:03^+^ (*n* = 4) and HLA‐B*45:01^+^ (*n* = 3) donors were stimulated with the NS1_195–203_ peptide to generate CD8^+^ T‐cell lines. The NS1_195‐203_ peptide immunogenicity was then assessed by an ICS assay (Figure 4). The NS1_195‐203_ peptide was the most immunogenic, as assessed by the level of IFNγ production, when presented by HLA‐B*44:03 (*n* = 2/4, average; 6.52%), and less by HLA‐B*44:02 (*n* = 2/4, average; 0.23%) (Figure 4b). A small level of IFNγ^+^CD8^+^ T‐cell was detected in HLA‐B*40:01^+^ samples (*n* = 3/4, average; 0.057%), but not for HLA‐B*40:02^+^ (*n* = 0/3) or HLA‐B*45:01^+^ (*n* = 0/3) samples tested (Figure 4b).

**Figure 5:**
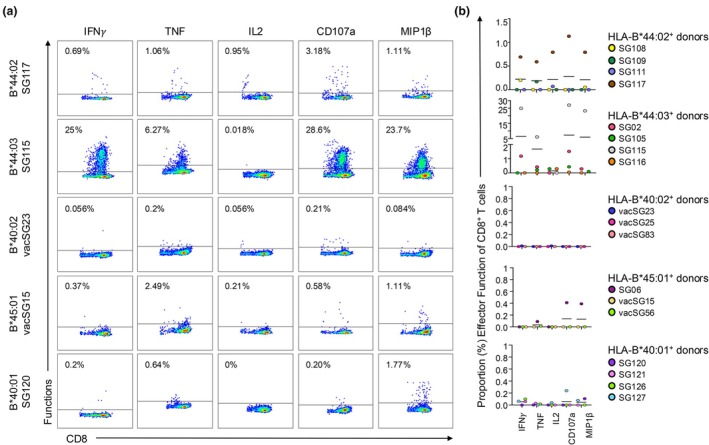
Summary of all cytokine and marker functions expressed. NS1_195–203_ specific CD8^+^ T‐cell lines derived from donors with HLA‐B*40:01 (*n* = 4), HLA‐B*40:02 (*n* = 3), HLA‐B*44:02 (*n* = 4), HLA‐B*44:03 (*n* = 4) and HLA‐B*45:01 (*n* = 3) were intracellularly stained for IFNγ, TNF, CD107a, IL2 and MIP1β after stimulation with either the NS1_195–203_ peptide, no peptide (negative control) or 500x (positive control). **(a)** Representative FACS plots of effector function production by CD8^+^ T cells from donors with HLA‐B*40:01, HLA‐B*40:02, HLA‐B*44:02, HLA‐B*44:03 and HLA‐B*45:01. **(b)** Summary of CD8^+^ T‐cell effector functions produced in response to NS1_195‐203_ stimulation (minus negative control) in donors with each of the B44 supertype molecules. Bars represent the average (mean).

**Figure 6:**
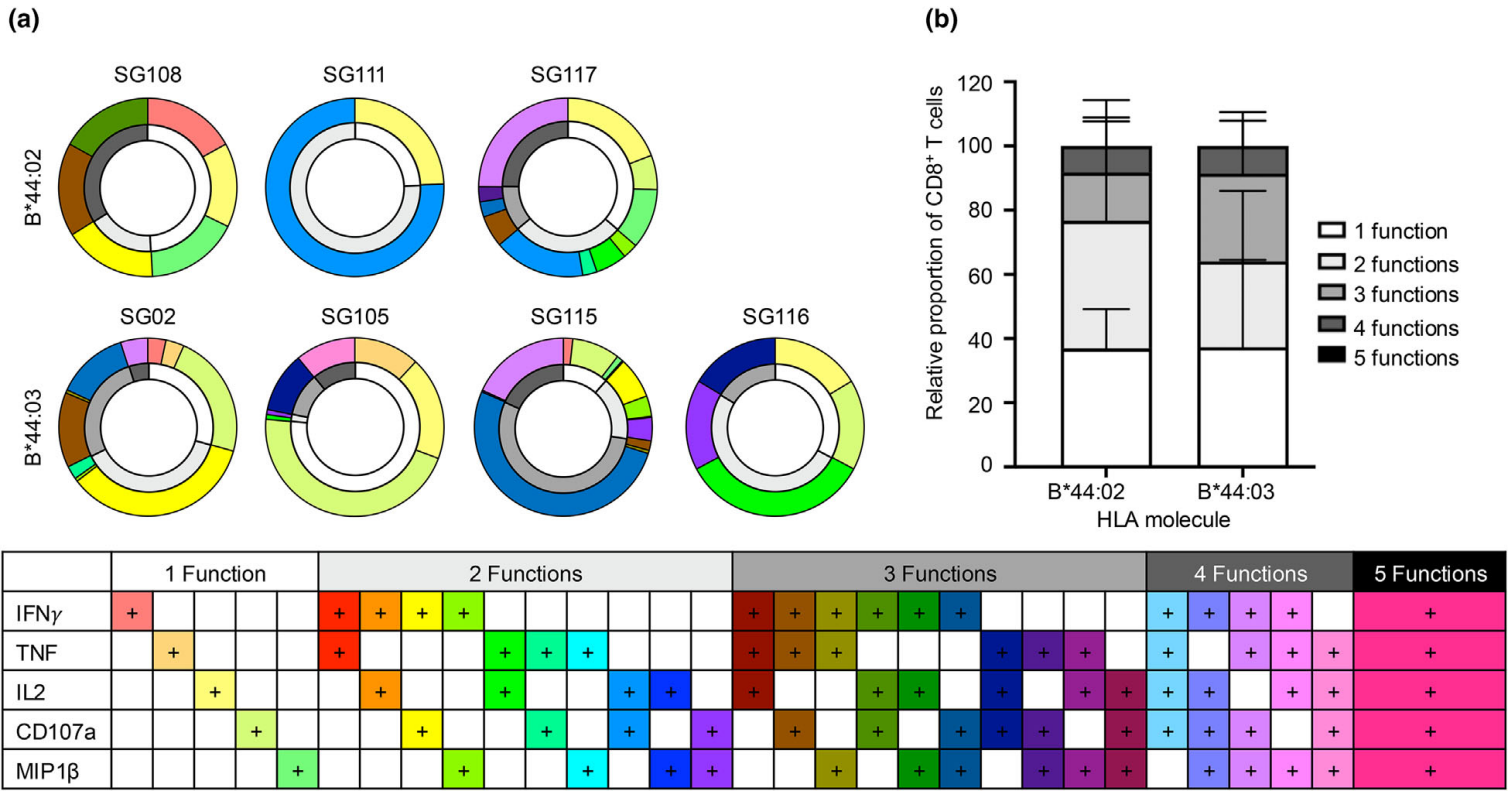
Polyfunctional CD8^+^ T‐cell responses of NS1_195–203_‐specific T‐cell lines derived from donors expressing HLA‐B*44:02 or HLA‐B*44:03. NS1_195–203_ specific CD8^+^ T‐cell lines derived from donors with HLA‐B*44:02 (*n* = 4) and HLA‐B*44:03 (*n* = 4) were stimulated with either NS1_195–203_ peptide, no peptide (negative control) or 500× (positive control). Production of IFNγ, TNF, CD107a, IL2 and MIP1β was assessed via intracellular cytokine staining. **(a)** Summary of CD8^+^ T‐cell effector functions minus the no peptide control, represented as a double‐ring donut where the inner ring shows the number of effector functions (colour white to black) and the outer ring shows the specific CD8^+^ T‐cell effector functions with each key defined in the table below. **(b)** Bar graph summarising the proportion of CD8^+^ T cells producing 1–5 effector functions. Lines and error bars are representative of the average and standard deviation. A two‐way ANOVA was also calculated, and no significance was reported.

Next, individual (Figure 5) and combinatorial (Figure 6) production of effector functions, including IFNγ, TNF, CD107a, IL2 and MIP1β from the ICS assay was analysed. A similar trend to the immunogenicity hierarchy defined by IFNγ production was observed for the other effector functions. The strongest responses were in CD8^+^ T‐cell lines derived from HLA‐B*44:03^+^ samples, where all samples produced at least one effector function but collectively minimal amounts of IL2 (average: 0.14%) were detected, while a higher percentage of TNF (average: 1.67%), CD107a (average: 7.2%) and MIP1β (average: 5.9%) was observed (Figure 5b). When assessed in combination, the majority of CD8^+^ T cells produced 1, 2 or 3 effector functions simultaneously, and some expressed 4, but none expressed all five effector functions (Figure 6). Similarly, the CD8^+^ T cells derived from *n* = 3/4 HLA‐B*44:02^+^ samples produced an average range of effector functions, including TNF (average: 0.19%), CD107a (average: 0.28%), MIP1β (average: 0.21%) and IL2 (average: 0.21%) (Figure 5b). In combination, the majority of NS1_195‐203_‐specific CD8^+^ T cells from HLA‐B*44:02^+^ samples produced 1, 2 or 3 effector functions, with only two samples making four simultaneously (Figure 6). There was no significant difference in the number of effector functions produced by CD8^+^ T cells derived from these samples expressing either of the two HLA‐I molecules (Figure 6). Again, limited to no effector functions were detected in CD8^+^ T cells derived from donors with the other HLA‐B44 supertype molecules.

## Discussion

HLA‐I molecules are essential for CD8^+^ T‐cell activation against viral pathogens.^2^, ^23^, ^24^ In the context of influenza, it has been well characterised that HLA‐I can present conserved peptides to T cells and may be well suited as a target for a universal influenza vaccine.^23^ However, one main challenge is that HLA‐I are highly polymorphic. At the time of writing, more than 28 000 *HLA‐I* alleles have been identified, and thus including peptides or even whole proteins which contain CD8^+^ T‐cell epitopes specific to each of these allomorphs is highly challenging.^10^, ^25^ In recent years, HLA‐I molecules have been categorised into HLA supertypes.^15^, ^16^, ^26^ HLA have been grouped into supertypes based on similar peptide binding motif,^16^ conservation of HLA residues in the B and F binding pockets,^15^ and more recently, during the course of this study, based on published pHLA‐I structures.^17^ In theory, it is possible that several HLAs within the same supertype could present the same peptide and activate CD8^+^ T cells. This has been seen with several influenza‐derived peptides, including the HLA‐A*03:01, HLA‐A*11:01 and HLA‐A*31:01‐restricted M2_45‐54_ and PA_104‐113_ peptides,^27^ the HLA‐B*07:02 and HLA‐B*35:01 restricted NP_418‐426_ peptide^28^ and the HLA‐A*01:01 and HLA‐A*26:01‐restricted NP_140‐148_.^2^, ^27^ Therefore, selecting peptides that can be presented by several HLA‐I allomorphs and induce strong CD8^+^ T‐cell responses for inclusion in a vaccine has the potential to cover significant proportions of the global population, and is an area that deserves further investigation.

To further explore this phenomenon, we chose the known immunogenic peptide NS1_195‐203_.^18^ This peptide has been described as immunogenic in one HLA‐B*44:03^+^ sample, leading to > 70% IFNγ^+^CD8^+^ T cells in a NS1_195‐203_‐specific CD8^+^ T‐cell line.^18^ In addition, the NS1_195‐203_ is conserved in A/H1N1 and A/H3N2 influenza viruses. HLA‐B*44:03 belongs to the HLA‐B44 supertype that includes HLA‐B*44:02, HLA‐B*40:01, HLA‐B*40:02 and HLA‐B*45:01.^15^

Using *in silico* TFold and AlphaFold2, we generated five separate predictive models of HLA‐B*40:01, HLA‐B*40:02, HLA‐B*44:02, HLA‐B*44:03 and HLA‐B*45:01 with the NS1_195‐203_ peptide. These methods provided a quick insight into observing the potential peptide‐HLA presentation for the HLA‐B44 supertype. Overall, the two predictive tools gave similar structures, at the exception of HLA‐B*44:03. The prediction scores from AlphaFold2 suggest that all five allomorphs would be able to present the NS1_195‐203_ peptide. However, TFold predictions were more confident that both HLA‐B*44:03 and HLA‐B*44:02 than the other allomorphs, with a score below 6.8.^20^ Interestingly, those two allomorphs also provided the strongest CD8^+^ T‐cell response observed in the samples tested. It is important to note that prediction models do not provide definitive proof that HLA‐B*40:01, HLA‐B*40:02 and HLA‐B*45:01 can effectively present the NS1_195‐203_ peptide. It does, however, provide an insight to peptide presentation and could allow for further research that can validate the results via experimental structure determination.

Interestingly, despite being predicted to present the NS1_195‐203_ peptide, variable CD8^+^ T‐cell responses were seen in cells with the different HLA‐I allomorphs. Half (*n* = 2/4) of our HLA‐B*44:02 and HLA‐B*44:03^+^ samples had positive CD8^+^ T‐cell responses towards the NS1_195‐203_ peptide, while several donors with HLA‐B*40:01 had weak CD8^+^ T‐cell responses. This result is unsurprising, as it is well established that not all donors will respond to all epitopes, even when they possess the ‘correct’ HLA‐I molecule.^29^, ^30^, ^31^, ^32^ This could be because of many factors,^33^, ^34^ including previous infection or vaccination history of an individual,^35^ the frequency of peptide‐specific naïve^36^, ^37^ or memory CD8^+^ T cells.^38^ The largest CD8^+^ T‐cell responses were seen in the cells derived from HLA‐B*44:03^+^ samples, consistent with the first known HLA‐I restriction for this peptide.^18^ Analysis of several cytokines revealed polyfunctional CD8^+^ T‐cell responses indicating that some individuals with these alleles may potentially be protected against A/H1N1 and A/H3N2 influenza viruses if vaccinated with the NS1_195‐203_ peptide. Conversely, limited to no CD8^+^ T‐cell responses were seen in cells derived from HLA‐B*40:02^+^ or HLA‐B*45:01^+^ individuals.

A better understanding of what exactly determines a presented peptide to be ‘immunogenic’ to CD8^+^ T cells, and as yet, there is no single correlating determinant. Aside from CD8^+^ T cell‐specific distinctions which influence the level of immunogenicity in any given donor, as discussed above, other factors such as the timing of protein production and abundance of protein during infection,^39^ peptide processing,^40^ peptide affinity for HLA‐I molecules^41^ or even the combination of other HLA‐I molecules within an individual^42^, ^43^ all have the potential to influence peptide presentation and subsequently CD8^+^ T‐cell activation.^33^ As such, what makes a peptide ‘immunogenic’ is likely to be multi‐faceted and challenging to predict, although new approaches using AI and machine learning may assist in the future.^44^ Currently, the grouping of HLA‐I supertypes is focused on peptide presentation by the HLA‐I molecules, with little to no consideration for peptide immunogenicity. Although extending prediction to peptide immunogenicity has great potential to improve the use of T cells in broader therapeutics. Thus, it is important to continue to assess immunogenicity in the context of HLA‐I supertype to contribute and build to this knowledge.

Consequently, while targeting HLA supertype allomorphs for vaccine development remains a possibility and may allow narrowing the breadth of peptides to be included, the current knowledge on peptide immunogenicity within HLA‐I supertype remains limited. However, new and additional immunogenic data as well as structures of different HLA‐I molecules could establish a greater degree of knowledge for the development of a universal T cell‐mediated vaccine in the future.

## Methods

### Ethics, donors and human peripheral blood mononuclear cells

Whole blood or buffy coats were obtained from voluntary donors or the Australia Red Cross Lifeblood, respectively. Peripheral blood mononuclear cells (PBMCs) were isolated using the Ficoll density gradient centrifugation method employed^8^, ^29^ and were also cryogenically stored at −80°C or in liquid nitrogen until required. PBMCs were HLA typed by CareDx Pty Ltd, (Fremantle, Western Australia; AlloSeq Tx) or the Victorian Transplant and Immunogenetics Service (VTIS). All research was approved by the La Trobe University Human Ethics Committee (approval no.: HEC21097) and undertaken in accordance with the Declaration of Helsinki.

### Generation of NS1_195–203_ specific CD8^+^ T‐cell lines

The NS1_195–203_ peptide (SETLQRFAW) was ordered at > 80% purity from GenScript (Piscataway, USA). PBMCs from HLA‐B*40:01^+^, HLA‐B*40:02^+^, HLA‐B*44:02^+^, HLA‐B*44:03^+^ and HLA‐B*45:01^+^ donors were stimulated with the NS1_195–203_ peptide to generate NS1_195–203_‐specific T‐cell lines as previously described.^45^ Briefly, one‐third of the PBMCs pulsed with 10 μm of the NS1_195–203_ in R0 (RPMI‐1640, Gibco, New York, USA supplemented with 1x non‐essential amino acids, 100× NEAA Gibco; 1× Penicillin Streptomycin Glutamine, Gibco; 5 mm HEPES, Sigma‐Aldrich, St. Louis, USA; 2 mm L‐glutamine, Sigma‐Aldrich and; 50 μm of β‐mercaptoethanol, Sigma‐Aldrich) and were incubated for 90 min at 37°C with 5% CO_2_. The NS1_195–203_ stimulated PBMCs were then washed twice in R0 and were added to the remaining two‐thirds unstimulated PBMCs in RF10 (R0 supplemented with foetal bovine serum; FBS, Bovogen, Melbourne, Australia). The T‐cell lines were then cultured for 10 days in RF10. On Days 3 to 4 and Day 7, 10 IU mL^−1^ of recombinant human IL2 (Peprotech, Rocky Hill, USA) was added.

### Intracellular cytokine staining of NS1_195_ _–203_ specific CD8 ^+^ T‐cell lines

NS1_195‐203_ specific CD8^+^ T‐cell lines were counted using the trypan blue exclusion method and 2 × 10^5^ cells were harvested for each experimental condition. NS1_195–203_ specific CD8^+^ T‐cell lines were stimulated with 10 μm peptide. Cell Stimulation Cocktail 500× (eBiosciences, San Diego, USA) as a positive control or no peptide negative control. All cell lines were then incubated for 5 h at 37°C with 5% CO_2_ in the presence of Golgi‐stop (1:1400; BD Biosciences, Franklin Lakes, USA), Golgi‐Plug (1:1000; BD Biosciences) and anti‐human CD107a‐AF488 (1:200; eBiosciences). After 5 h, cells were stained with anti‐human CD3‐BV480 (1:100; BD Biosciences), anti‐human CD4‐BV650 (1:100; BD Biosciences), anti‐human CD8‐PerCPCy5.5 (1:50; BD Biosciences), anti‐human CD14‐APCH7 (1:100; BD Biosciences), anti‐human CD19‐APCH7 (1:100; BD Biosciences) and Live/Dead‐NIR (1:1000; Molecular Probes, Eugene, USA) for 30 min at 4°C. Following this, all cells were washed with PBS and fixed and permeabilised with BD‐Fix Perm buffer (BD Biosciences) for 20 min at 4°C. Finally, cells were intracellularly stained with anti‐human IFNγ‐BV421 (1:100; BD Biosciences), anti‐human MIP1β‐APC (1:100; BD Biosciences), anti‐human TNF‐PeCy7 (1:100; BD Biosciences) and anti‐human IL2‐PE (1:50; BD PharMingen, Franklin Lakes, USA) for 30 min at 4°C. Cells were washed and acquired on a BD FACSymphony A3 analyser (BD Biosciences) and analysed using FlowJo version 10.10.0 (BD Biosciences). Two‐way ANOVA calculations and figures were calculated and graphed using Prism v10.3.1 (GraphPad Software, Boston, USA). Samples are gated as per Supplementary figure 4.

### Predicted binding affinity of NS1_195_ _–203_ binding to each of the HLA‐B44 supertype molecules

NetMHCpan‐4.1 was used to predict the affinity of the NS1_195‐203_ peptide (SETLQRFAW) binding to the HLA‐B44 supertype molecules comprising HLA‐B*44:02, HLA‐B*44:03, HLA‐B*40:02, HLA‐B*45:01 and HLA‐B*40:01.^19^ The threshold for a strong peptide binder was < 0.5, a weak peptide binder was from 0.5 to 2, and a peptide non‐binder was > 2.^19^

### Protein sequence alignment of the B44 supertype molecules

The protein sequences for each of the HLA‐B44 allomorphs were downloaded from the IPD‐IMGT/HLA database^10^ and were aligned using UniProt Align.^46^

### TFold and AlphaFold2 structural analysis of NS1_195_ _–203_ peptide to each HLA‐B44 supertype molecule

To predict peptide‐HLA structures we used the TFold pipeline available on https://github.com/v‐mikhaylov/tfold‐release.^20^ The source code was implemented on a local computer. AlphaFold protein models were generated using AlphaFold2 Multimer via the ColabFold interface on Google Colab.^47^ Default settings were used, with sequence alignments created by MMseqs2 and final models underwent structural relaxation with AMBER on the Colab platform.

## Conflict of interest

The authors declare no conflict of interest.

## Supporting information


Supplementary figures 1‐4

## Data Availability

The data that support the findings of this study are available from the corresponding authors, EJG and SG, upon reasonable request.
