# Supplementary material for: Lack of immunogenicity for an influenza‐derived peptide across the HLA‐B44 supertype molecules
Source: Clin Transl Immunology. 2025 Sep 19;14(9):e70051. doi: 10.1002/cti2.70051 (PMC12447248; doi:10.1002/cti2.70051)
Supplement: Supplementary file 1 — Supplementary figures 1‐4 [file CTI2-14-e70051-s001.pdf]

Supplementary Figure 1

```
                    #
HLA-B*44:03 GSHSMRYFYTAMSRPGRGEPRFITVGYVDDTLFVRFDSDATSPRKEPRAPWIEQEGPEYW 60
HLA-B*44:02 GSHSMRYFYTAMSRPGRGEPRFITVGYVDDTLFVRFDSDATSPRKEPRAPWIEQEGPEYW 60
HLA-B*45:01 GSHSMRYFHTAMSRPGRGEPRFITVGYVDDTLFVRFDSDATSPRKEPRAPWIEQEGPEYW 60
HLA-B*40:01 GSHSMRYFHTAMSRPGRGEPRFITVGYVDDTLFVRFDSDATSPRKEPRAPWIEQEGPEYW 60
HLA-B*40:02 GSHSMRYFHTSVSRPGRGEPRFITVGYVDDTLFVRFDSDATSPRKEPRAPWIEQEGPEYW 60
            ********:*::************************************************

                            #  ##            #
HLA-B*44:03 DRETQISKTNTQTYRENLRTALRYYNQSEAGSHIIQRMYGCDVGPDGRLLRGYDQDAYDG 120
HLA-B*44:02 DRETQISKTNTQTYRENLRTALRYYNQSEAGSHIIQRMYGCDVGPDGRLLRGYDQDAYDG 120
HLA-B*45:01 DRETQISKTNTQTYRESLRNLRGYYNQSEAGSHTWQRMYGCDLGPDGRLLRGYNQLAYDG 120
HLA-B*40:01 DRETQISKTNTQTYRESLRNLRGYYNQSEAGSHTLQRMYGCDVGPDGRLLRGHNQYAYDG 120
HLA-B*40:02 DRETQISKTNTQTYRESLRNLRGYYNQSEAGSHTLQSMYGCDVGPDGRLLRGHNQYAYDG 120
            ****************.**.   **********  * *****:*********::* ****

                                  #   #
HLA-B*44:03 KDYIALNEDLSSWTAADTAAQITQRKWEAARVAEQLRAYLEGLCVESLRRYLENGKETLQ 180
HLA-B*44:02 KDYIALNEDLSSWTAADTAAQITQRKWEAARVAEQDRAYLEGLCVESLRRYLENGKETLQ 180
HLA-B*45:01 KDYIALNEDLSSWTAADTAAQITQRKWEAARVAEQDRAYLEGLCVESLRRYLENGKETLQ 180
HLA-B*40:01 KDYIALNEDLRSWTAADTAAQISQRKLEAARVAEQLRAYLEGECVEWLRRYLENGKDKLE 180
HLA-B*40:02 KDYIALNEDLRSWTAADTAAQITQRKWEAARVAEQLRAYLEGECVEWLRRYLENGKETLQ 180
            ********** ***********:*** ******** ****** *** *********:.*:
```

Supplementary Figure 1. Protein sequence alignment of the peptide binding cleft of the HLA-B44 supertype molecules. The sequences of the HLA-B44 supertype molecules were downloaded from the IPD-IMGT/HLA database [10] and aligned using UniProt Align. [46] Residues 1 – 180 of the HLA that compose the peptide binding cleft are shown with variations in the amino acid sequence highlighted in yellow. The orange hashtag highlights polymorphic residue within the HLA B pocket, and the cyan hashtags highlight polymorphic residues within the HLA F pocket.

Supplementary Figure 2

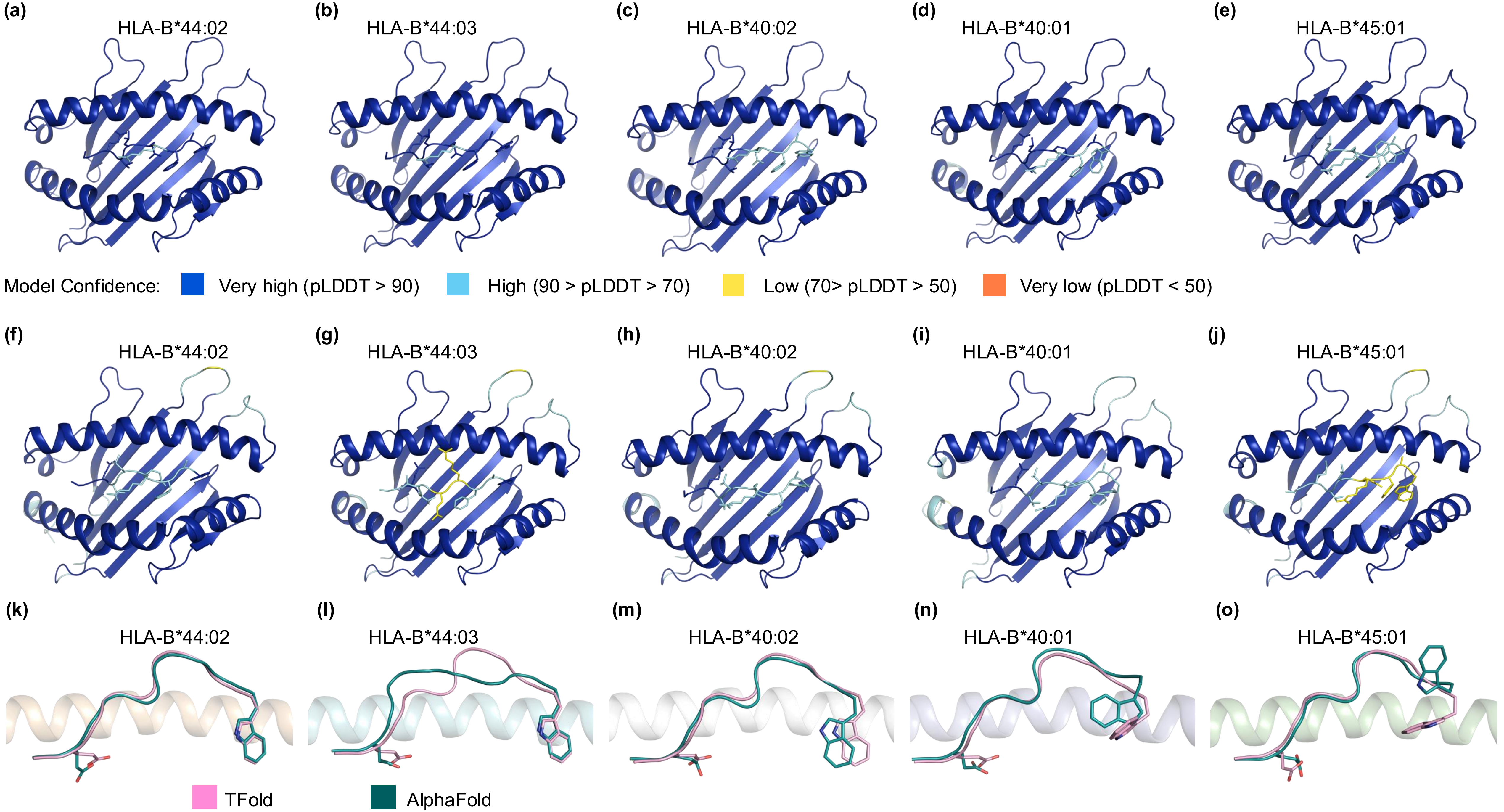

Supplementary Figure 2. Predicted structures for HLA-B44 supertype molecules presenting the $NS1_{195–203}$ peptide. **(a-e)** TFold-generated models are displayed using the default AlphaFold2 colour coding based on model confidence with a gradient from blue for high confidence to red for low confidence. **(f-j)** AlphaFold2-generated models for each HLA supertype are shown with the same colour coding as panels (a-e). **(k-o)** Superimposition of the TFold and AlphaFold2 predicted models for each supertype. Each HLA is represented as a cartoon, the TFold predicted peptide is in pink and the AlphaFold2 predicted peptide in teal. The P2-Glu and P9-Trp side chains are represented as sticks.

Supplementary Figure 3

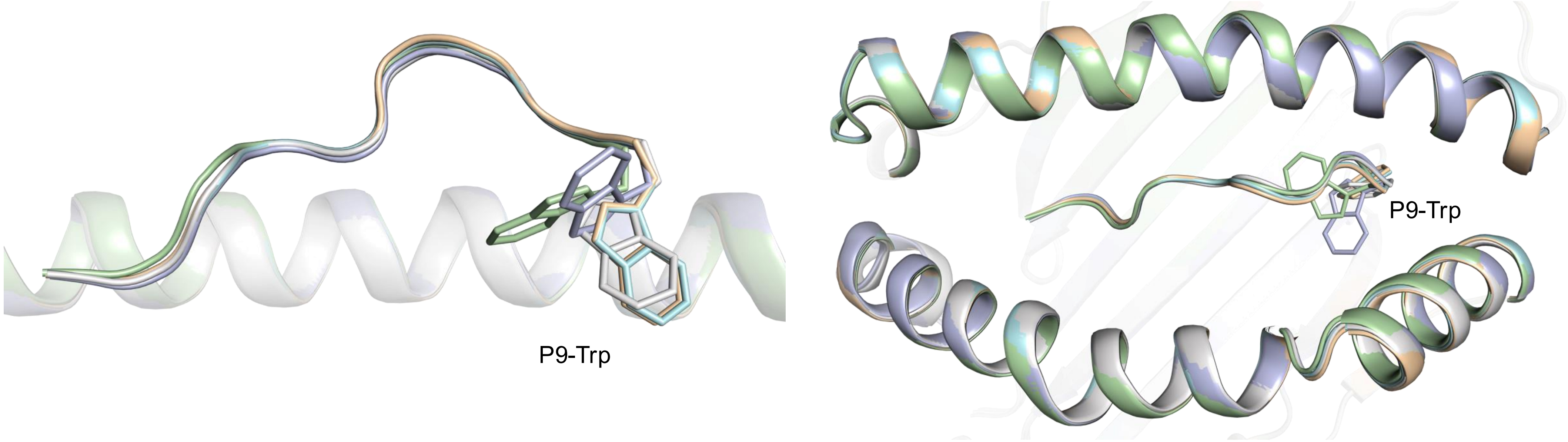

Supplementary Figure 3. P9-Trp conformations in each HLA-B44 supertype. The $NS1_{195–203}$ peptide of each HLA allomorph was aligned to show the different side-chain conformations of P9-Trp. Each HLA (in cartoon) and the bound peptide (in sticks) are coloured by HLA: HLA-B*44:02 in orange, HLA-B*44:03 cyan, HLA-B*40:02 in grey, HLA-B*40:01 in light blue and HLA-B*45:01 in green. The panels show a side and top view.

Supplementary Figure 4

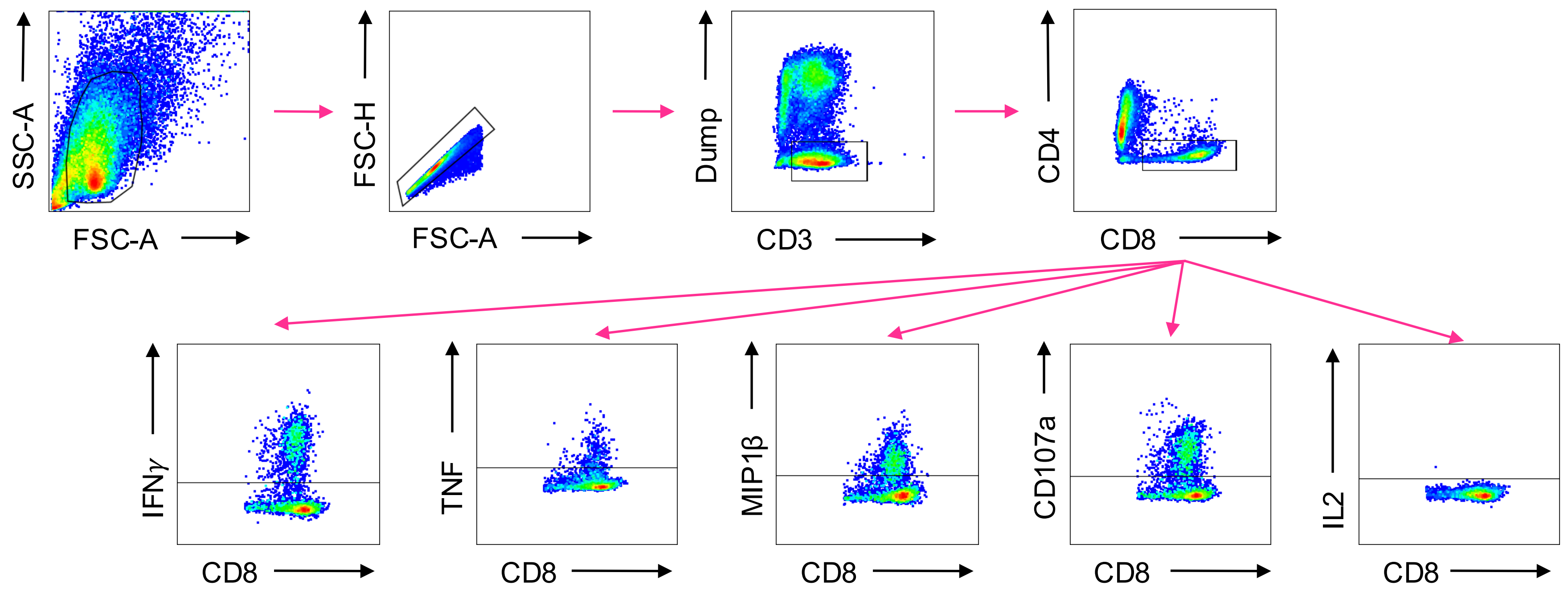

Supplementary Figure 4. Gating strategy for the assessment of CD8⁺ T cell responses. Representative FACS plots from sample SG115 showing the Gating strategy used for the analysis of all intracellular cytokine staining assays measuring CD8⁺ T cell function. Cells are gated on lymphocytes, single cells, live CD3mid-high, CD8mid-high T cells and then effector function. CD8⁺ IFNγ⁺ cells are represented in Figure 4. CD8⁺ T cells producing IFNγ, TNF, MIP1β, IL2 or CD107a are represented in Figure 5. Boolean gating was used to determine CD8⁺ T cell polyfunctionality in Figure 6.
